# Neonatal pain management: still in search of the Holy Grail 

**DOI:** 10.5414/CP202561

**Published:** 2016-04-18

**Authors:** Karel Allegaert, John N. van den Anker

**Affiliations:** 1 Neonatal Intensive Care Unit, University Hospitals and Department of Development and Regeneration, KU Leuven, Belgium,; 2 Intensive Care and Department of Surgery, Erasmus MC-Sophia Children’s Hospital, Rotterdam, The Netherlands,; 3 Division of Paediatric Pharmacology and Pharmacometrics, University Children’s Hospital Basel, Switzerland, and; 4 Division of Pediatric Clinical Pharmacology, Children’s National Medical Center, Departments of Pediatrics, Integrative Systems Biology, Pharmacology & Physiology, George Washington University School of Medicine and Health Sciences, Washington, DC, USA

**Keywords:** newborn, pain, pain management, developmental pharmacology, pharmacogenetics

## Abstract

Inadequate pain management but also inappropriate use of analgesics in early infancy has negative effects on neurodevelopmental outcome. As a consequence, neonatal pain management is still in search for the Holy Grail. At best, effective pain management is based on prevention, assessment, and treatment followed by a re-assessment of the pain to determine if additional treatment is still necessary. Unfortunately, epidemiological observations suggest that neonates are undergoing painful procedures very frequently, unveiling the need for effective preventive, non-pharmacological strategies. In addition, assessment is still based on validated, multimodal, but subjective pain assessment tools. Finally, in neonatal intensive care units, there is a shift in clinical practices (e.g., shorter intubation and ventilation), and this necessitates the development and validation of new pharmacological treatment modalities. To illustrate this, a shift in the use of opioids to paracetamol has occurred and short-acting agents (remifentanil, propofol) are more commonly administered to neonates. In addition to these new modalities and as part of a more advanced approach of the developmental pharmacology of analgesics, pharmacogenetics also emerged as a tool for precision medicine in neonates. To assure further improvement of neonatal pain management the integration of pharmacogenetics with the usual covariates like weight, age and/or disease characteristics is needed.

## Introduction: on how we act and what we know 

Inadequate pain management in neonatal life impairs neurodevelopmental outcome. It alters pain thresholds, physiological responses and stress- or pain-related behavior beyond early infancy [1, 2, 3, 4]. Therefore, pain management in neonates should not just be driven by ethics or empathy but it should be viewed as part of normal medical and nursing care. At the same time, there are emerging animal experimental and human epidemiological data that show an association between the exposure to analgesics and impaired neurodevelopmental outcome [5, 6, 7]. As a consequence, the management of neonatal pain remains in search of a new balance because these conflicting observations are the main drivers to tailor our current practices. To reach this new balance new treatment modalities, including non-pharmacological and pharmacological strategies, will need to be developed and validated [7, 8, 9]. 

### Epidemiology 

An additional argument in support of the need for new treatment modalities, including non-pharmacological interventions, is that epidemiological observations provide evidence for shifts in neonatal pain management practices [10]. In Table 1, we summarized observations on pain management practices in (pre)term neonates in the first 14 days of postnatal life [11, 12, 13]. Compared to the cohorts of 2001 and 2005 – 2006, the Rotterdam group (2009) documented a statistically significant, but clinically modest decrease in the number of painful procedures (11 instead of 14/day) [11, 13], with more routine use and integration of non-pharmacological interventions, and a decrease in opioid use in ventilated newborns [13]. 

**Pain management in neonates is not only limited to pharmacological interventions**

Non-pharmacological interventions stress the fact that not only what, but also how we perform painful procedures matters [8, 9, 14]. The focus needs to be on less invasive techniques, preventive strategies, or complementary techniques [14, 15]. Environmental (light or noise exposure), behavioral (positioning, handling, swaddling), and non-pharmacological (sucrose, breastfeeding, pacifier, non-nutritive sucking) interventions prevent, alleviate, or even eliminate pain. Adaptations of procedural practices may be a very powerful approach to prevent pain. Such strategies also cover the use of central venous catheters instead of multiple peripheral perfusions, individualized monitoring techniques (blood pressure measurement interval, vital signs registration), adapted nursing techniques (e.g., frequency of endotracheal suctioning, skin and wound care, tape and wound dressing) or promoting skin-to-skin contact between newborn and parents [8, 9, 14, 15]. 

**Assessment is still subjective and thus suboptimal**

The absence of verbalization is very likely one of the most important thresholds for the proper diagnosis and management of neonatal pain [16, 17, 18]. Pain in the newborn is not easily recognized and remains commonly under- or untreated. Pain assessment is still based on validated, multimodal, but subjective pain assessment tools, like the COMFORTneo score [17]. Until more advanced equipment becomes available to quantify pain, we should apply such a validated pain assessment tool in clinical practice and train health care providers in using these tools in a standardized way to guarantee a reasonable interobserver variation in the assessment of pain in neonates [18, 19]. 

## Developmental pharmacology of analgesics in neonates: a moving target 

The general paradigm on pain management in neonates is driven by assessment, titration of a given intervention (non-pharmacological, pharmacological, or both) and subsequent re-assessment [3, 8]. Clinical pharmacology aims to predict (side) effects of compound specific pharmacokinetics (PK) and pharmacodynamics (PD) at the level of the population or – preferably – the individual. These general concepts of clinical pharmacology obviously also apply to analgesics in neonates, but their maturational physiology and the associated variability within the population warrant a tailored approach [20, 21, 22]. PK in early infancy display extensive intra- and inter-individual variability. This can in part be explained by e.g., maturational changes in body composition, protein binding, and compartment sizes during infancy. Similar, drug-related metabolic processes mature in an enzyme-specific pattern while renal function also displays an age-dependent increase in clearance. From a clinical pharmacology perspective, the consequence of such a dynamic setting is extensive variability in early infancy [20, 21, 22]. 

This dynamic setting further stresses the need to perform focused studies on new analgesics introduced in the neonatal unit [22]. Moreover, it also means that new covariates emerging in other populations such as pharmacogenetics may further explain in part the variability observed in neonates, but need to be integrated with other maturational covariates like weight or age. We will first discuss the PK/PD of intravenous paracetamol and two short acting compounds (remifentanil, propofol) since these drugs have recently been introduced in neonatal intensive care units. Then we will provide guidance on how to integrate pharmacogenetics as another covariate to improve the predictability of individual PK or PD in early infancy. 

### Shift from opioids to paracetamol 

**Figure 1.**
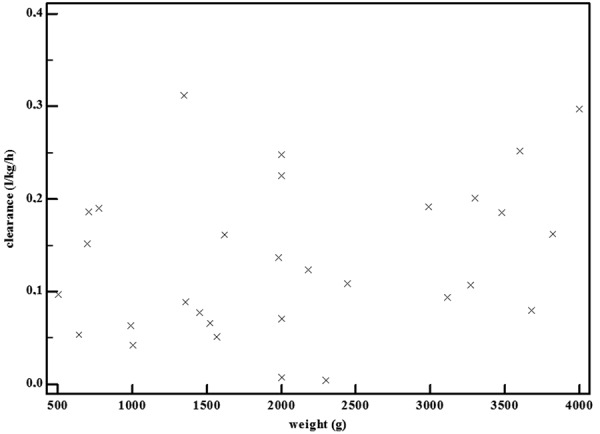
Individual clearance (L/kg/h) estimates in 30 (pre)term neonates following a single intravenous propacetamol (10 – 20 mg/kg paracetamol equivalent) administration show a modest increase with increasing weight (median clearance < 2 kg 0.123 to 0.16 L/kg/h in cases > 2 kg) (X-axis: weight, in g; Y-axis: paracetamol clearance, in L/kg/h) [28].

In an attempt to avoid or reduce opioid exposure, a shift to administer paracetamol has occurred, hugely facilitated by the availability of an intravenous formulation [23, 24, 25, 26, 27]. Despite the fact that intravenous paracetamol is still used off label in specific subpopulations (e.g., limited to term neonates, or, under 2 years of age in the United States), these formulations are increasingly used in (pre)term neonates [23, 26]. In adults, paracetamol is metabolized by the liver to paracetamol-glucuronide (47 – 62%) and paracetamol-sulphate (25 – 36%), and subsequently eliminated by the renal route. Only 1 – 4% is excreted unchanged in urine, and ~ 8 – 10% of paracetamol is oxidized to 3-hydroxy-paracetamol and the (hepatic) toxic metabolite N-acetyl-p-benzoquinone-imine (NAPQI). In neonates, paracetamol clearance is lower, and its variability is mainly explained by weight. This is further illustrated in Figure 1, where we plotted the individual paracetamol clearance estimates for 30 (pre)term neonates following a single intravenous paracetamol administration [28]. Similarly, also the formation clearance to paracetamol-glucuronide and paracetamol-sulphate, and primary renal elimination of paracetamol is mainly related to weight [29]. However, in contrast to the observations in adults, sulphation and not glucuronidation is the most important route of elimination in neonates [29]. 

The currently available evidence on the efficacy of paracetamol as analgesic supports the use of paracetamol for minor to moderately severe pain syndromes in early infancy [30]. Moreover, paracetamol has a clinically relevant opioid-sparing effect (66%) in neonates after major non-cardiac surgery [24]. In contrast, there is only a very limited analgesic effect of paracetamol when used for procedural (e.g., heel lancing) pain relief [23]. Short-term tolerance (hepatic, hemodynamics) has been described [31, 32], indicating no signs of hepatic intolerance during and after repeated administrations of intravenous paracetamol [31]. In contrast to the negative hemodynamic effects in adult intensive care patients, hemodynamic side effects of intravenous paracetamol in neonates are very modest [32]. Besides these short-term outcome side effects, there are some population-specific side effects that warrant focused attention. Recent epidemiological data showed a possible link between the (over) use of paracetamol during pregnancy or early infancy and immune deviations or neurodevelopmental problems (e.g., autism) [23]. Causality is still very questionable and for sure not yet proven, but pharmacovigilance is warranted to explore the potential association between paracetamol exposure and these outcome variables. 

### The need to generate knowledge of short-acting agents in neonates 

Taking the shift towards less invasive neonatal care (e.g., insure procedure, i.e., intubation – surfactant administration – extubation instead of prolonged mechanical ventilation) into account, remifentanil and propofol have been introduced in neonatal intensive care [2, 8, 9, 33, 34]. 

Remifentanil hydrochloride is a short-acting, µ-receptor opioid agonist. It achieves its peak analgesic effect within a minute of administration, 3 – 4 times faster when compared to fentanyl and much faster when compared to morphine [33]. Remifentanil undergoes metabolic clearance by plasma esterases, resulting in predictable clearance, irrespective of renal or liver dysfunction, and the plasma esterase activity is already quite mature at birth [35]. Its pharmacological profile seems suited for short procedural analgesia [36, 37, 38, 39, 40]. In neonates, this compound has mainly been used for short procedures like endotracheal intubation, laser surgery for retinopathy of prematurity or for insertion of a percutaneous intravenous central catheter with anecdotal experience during major surgery or mechanical ventilation [36, 37, 38, 39, 40]. Because of its rapid clearance, clinicians must be aware that the analgesic effects disappear very soon after discontinuation of remifentanil [33]. Other issues to consider are potential hyperalgesia, fast appearance of tolerance, and the risk of chest rigidity [33]. 

Propofol (2,6 di-isopropylphenol) is a highly lipophilic compound [34]. Propofol exhibits rapid distribution to the subcutaneous fat and the central nervous system compartments with subsequent redistribution to the blood compartment and metabolic clearance [34, 41]. Because propofol is not soluble in water, propofol clearance is exclusively through metabolic clearance [41, 42]. Although multiple hepatic and extrahepatic human cytochrome (CYP) P450 isoforms (hydroxylation, mainly CYP2B6) are involved in propofol metabolism, glucuronidation is the major metabolic pathway after a single intravenous bolus in adults [41, 42, 43]. In contrast, propofol clearance in neonates is much lower, and mainly driven by postnatal age [43]. Interestingly and based on 24 hours urine collections, not glucuronidation but hydroxylation is the most important route of propofol metabolism in the first 10 days of postnatal life [42, 43]. In neonates, there is reported experience with intravenous bolus propofol administration to facilitate endotracheal intubation, but there is important variability in clinical characteristics, outcome criteria, comedication and doses evaluated in the different studies [34, 44, 45]. Similarly, there is conflicting information on the magnitude of hemodynamic (side) effects of propofol in (pre)term neonates. In a recent Cochrane review, Shah et al. [34] concluded that regarding the use of propofol in neonates, no practice recommendations could be made yet. 

### How to integrate pharmacogenetics into developmental pharmacology of analgesics 

The emerging field of pharmacogenetics as a tool for personalized medicine – including pain management – reflects the notion that a specific (side) effect is not at random distributed in a specific population [46]. This obviously also holds promises for personalized pain management in young infants, but in addition to the usual covariates like weight, age and/or disease characteristics [22, 47]. We hereby strongly recommend the integration of pharmacogenetics as an additional covariate to improve individual PK or PD predictions in early infancy [22, 47]. In this special issue on various aspects of pain and its management, pharmacogenetics is repeatedly suggested to contribute to the PK/PD variability of analgesics. We will illustrate the complex interaction between maturational changes and polymorphisms comparing propofol and tramadol PK observations in early infancy. In essence, the utility of pharmacogenetics as predicting covariate is limited to periods during development in which genotype-phenotype concordance already exists [22, 47]. To illustrate this, a literature search on specific genetic polymorphisms related to PK/PD of analgesics in adults has been performed. Based on the findings of this search the relevance of specific genetic polymorphisms for optimizing analgesia in neonates will be explored. 

### Maturational drug metabolism and polymorphisms: is “concordance” already present? 

Besides age or weight as maturational covariates, genetic polymorphisms in drugmetabolizing enzymes, transporters or receptors may further contribute to the variability in PK/PD of analgesic drugs in early infancy if concordance already exists [23, 48]. Concordance is hereby restricted to the presence of a phenotype-genotype linkage similar to what is known in adults. Sufficient maturational driven phenotypic activity is needed before an impact of polymorphisms can be explored. We therefore try to illustrate this by comparing available observations on propofol and tramadol disposition [41, 42, 49]. 

Glucuronidation is the major metabolic pathway after a single intravenous bolus of propofol in adults. As a consequence, UGT1A9 promotor polymorphisms (UGT1A9-331C/T) affect propofol clearance in adults [41]. However, this can’t be directly translated to the neonate because not glucuronidation, but hydroxylation is the major route of propofol metabolic clearance in early neonatal life [43]. These in-vivo observations are in line with the available in vitro observations on UGT1A9 ontogeny describing a progressive increase in activity throughout infancy [50]. 

In contrast, concordance for the impact of cytochrome P450 enzyme 2D6 (CYP2D6) polymorphism on tramadol metabolism in early life has been described. Tramadol is a racemic mixture of two enantiomers, (+)-tramadol and (–)-tramadol hydrochloride [46, 49]. The analgesic effects of tramadol are mediated through noradrenaline re-uptake inhibition, increased serotonin release and decreased serotonin re-uptake in the spinal cord. Tramadol itself also has a weak µ-opioid receptor effect. Tramadol (M) is metabolized by cytochrome P450 enzyme 2D6 (CYP2D6) to the active metabolite (µ-opioid receptor agonist) O-desmethyltramadol (M1) and the inactive metabolite N-desmethyltramadol (M2) through cytochrome P450 3A4 and 2B6 (CYP3A4 and CYP2B6) [46, 49]. In adults, phenotypic M1 formation is more pronounced compared to M2 formation, but this ratio depends on CYP2D6 polymorphisms, quantified by the CYP2D6 activity score. Consequently, CYP2D6 polymorphisms result in differences in analgesia through differences in M1 formation. Besides CYP2D6 polymorphisms, variability in M1 disposition has been linked to maturational changes (weight, age), comedication (e.g., drug-drug interactions) or comorbidity (e.g., renal impairment) [46, 49]. 

**Figure 2.**
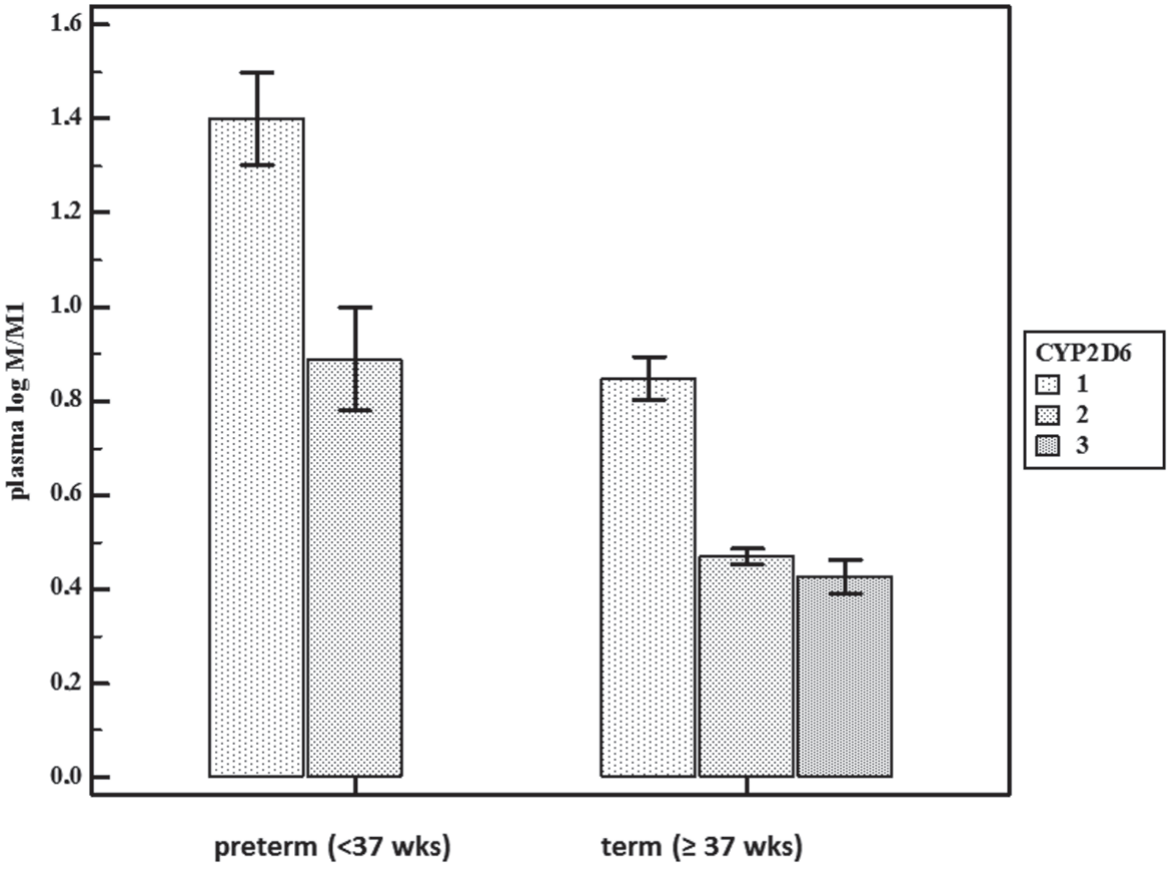
The impact of age (preterm (< 37 weeks) or term) and the CYP2D6 activity score (either 1, 2, or 3) on the plasma log M/M1 value. A lower M/M1 value hereby reflects a higher CYP2D6 activity, depending on both age and genetic polymorphisms. Individual plasma log M/M1 values were extracted from an earlier published dataset on tramadol disposition in neonates. There were no cases with a CYP2D6 activity score of 3 in the preterm age cohort. (M = tramadol; M1 = O-desmethyltramadol) (X-axis: preterm (< 37 week) or term (≥ 37 week) cases, Y-axis: plasma log M/M1 value) [49].

The complex interaction between maturational changes and CYP2D6 polymorphisms in early infancy has been illustrated in a dataset of 57 cases exposed to continuous intravenous tramadol [46, 49]. In this dataset, there was a significant decrease in plasma log M/M1 with an increasing CYP2D6 activity score, reflecting higher phenotypic CYP2D6 activity. In a forward multiple regression model, it was concluded that postmenstrual age and CYP2D6 polymorphisms determined O-demethylation activity in (pre)term neonates and young infants. Figure 2 illustrates the impact of both age (preterm vs. term cases) and CYP2D6 activity score (either 1, 2, or 3) on the plasma log M/M1 values, based on the earlier mentioned dataset in young infants [46, 49]. 

The absence of concordance for propofol but its presence for tramadol reflect different patterns of maturational activity of specific isoenzymes with a much more delayed phenotypic activity for glucuronidation (UGT1A9) as compared to demethylation (CYP2D6). The utility of pharmacogenetics as predicting covariate is hereby limited to periods during development in which genotype-phenotype concordance already exists, and is mainly driven by observations initially reported in adults. 

### Are polymorphisms linked to the PK/PD of analgesics in adults already of relevance in perinatal life? 

In this section we like to provide an overview of the impact of specific genetic polymorphisms linked to the PK/PD of analgesics in adults. Based on the findings of this search, the available evidence on the relevance of specific genetic polymorphisms to optimize analgesia in neonates will be explored. We will hereby consider polymorphisms related to drug metabolizing enzymes, drug transporters, and drug targets. 

**Drug-metabolizing enzymes**

Inter-individual variability exists in both phase I and phase II drug metabolism, and this variability can in part be explained by genetic polymorphisms. It may result in differences in either effects (e.g., concentrations, level of analgesia) or sideeffects (e.g., sedation, toxicity). 

The tramadol illustration earlier mentioned hereby provides the impact of CYP2D6 polymorphism on metabolic clearance to M1 in neonates, in line with similar observations in adults for tramadol or codeine [46, 49]. In the specific setting of breastfeeding, maternal CYP2D6 ultrafast metabolizer status – especially when combined with UGT2B7 *2/*2 polymorphism – results in higher exposure and an increased risk for central nervous system depression [51]. In contrast, CYP2C8 and CYP2C9 polymorphisms could not explain the variability in patent ductus closure during ibuprofen administration in preterm neonates [52]. Similarly, CYP2C8 and CYP2C9 polymorphisms had no effect on the relative infant dose (all < 1%) through breastfeeding in mothers treated with ibuprofen [53]. An association between CYP3A5 polymorphisms (higher drug metabolism) and paracetamol-induced liver toxicity has been described in adults [54]. This is unlikely in neonates, because the overall phenotypic CYP3A5 in early infancy is much lower [20, 21, 22]. 

If we further focus on phase II processes, Matic et al. [55] recently described the impact of UGT2B7 polymorphisms on the morphine-3-glucuronide/morphine ratio following a single bolus administration of morphine (0.3 mg/kg) in preterm neonates. Since sulphation activity is already more prominent, it seems more appropriate to explore these polymorphisms. Sulphotransferase enzymes catalyze sulphate conjugation, including paracetamol sulphation. As explored by Leeder et al. [48], exposure to paracetamol during pregnancy has been associated with a modest increase in the risk (RR 1.5 – 1.7) of gastroschisis in the infant. Based on in-vitro liver cytosol preparations, it has been suggested that paracetamol sulphation by the fetus might be linked with this increased incidence of gastroschisis. Clearly, further investigations into the genetic variability in both maternal (SULT1A1, SULT2A1) and fetal (SULT1A3/4) sulfation might shed more light on this association [48]. 

**Drug transporters**

Membrane transporters are crucial in the transport of compounds, and subsequently also mediate the uptake, regional distribution, and excretion of different compounds, including analgesics and its metabolites. Compared to the knowledge on the ontogeny of drug metabolizing enzymes, data on the maturation of human drug transporter expression and activity is still much more limited, but was recently summarized [47]. In fact, these authors also suggest to explore pharmacogenetic concordance in early infancy and other age cohorts to learn more about their ontogeny. 

The maturational changes in expression and activity of P-glycoprotein (P-gp) may affect the ability of the neonate to efflux opioids from the central nervous system compartment back to the systemic circulation. This may in part explain the higher sensitivity to central nervous system depressive effects of opioids in neonates. To quantify P-gp expression and its ontogeny, post-mortem samples (20 weeks fetal age to adults) were immunostained for P-gp in endothelial cells of the blood-brain barrier [56]. The authors hereby documented a maturational expression in P-gp to reach adult levels beyond 3 months of age. This ontogenic pattern fits quite well with the observations that P-gp polymorphisms (ABCB1 polymorphism rs9282564) were associated with a higher risk of opioid-related respiratory depression in children, but not anymore in adults, and not yet in neonates [57]. Adding one copy of the minor allele increased the odds of prolonged stay to the respiratory depression 4.7 fold. This suggests extensive placental P-gp expression, confirmed by the observation that fetal P-gp polymorphisms affect fetal growth and birth weight [58]. Finally, and to illustrate the complex interaction between different covariates, Sistonen et al. [59] documented that a genetic model combining the maternal risk genotypes in CYP2D6 and P-gp was significantly associated with central nervous system depression in infants (OR 2.68) and their mothers (OR 2.74) during maternal codeine intake. 

**Drug targets**

Similar to drug transporters, the knowledge on the ontogeny of drug receptors or drug targets is very limited. Again, a search for concordance in early infancy for polymorphisms earlier documented in adults hereby provides clinical information on the phenotypic expression and activity. In the field of analgesia, we retrieved mainly reports on polymorphisms of the µ-opioid receptor (OPRM1), sometimes in combination with catechol-O-methyl transferase (COMT) polymorphisms. The combination of both (OPRM1/COMT) results in synergistic effects since the need for rescue morphine in mechanically ventilated newborns was associated with both polymorphisms, resulting in an OR of 5.12 in the OPRM1/COMT high-risk genotype [60]. This is line with findings reported in children following adenotonsillectomy, since COMT polymorphisms also played a significant role in the variation in postoperative pain perception and postoperative morphine requirements in children [61]. 

### Clinical practice and research: in search for the Holy Grail 

Effective pain management remains an important indicator of the quality of care provided to neonates, but observations on neuroapoptosis and integration of newer techniques and compounds force caregivers to reconsider the clinical and research aspects of “effective” pain management. 

In the clinical setting, a structured approach is needed. This is because there is still a gap between what we know and how we act [19, 62]. An effective approach (e.g., evidence-based practice for improving quality (EPIQ) initiative) has been described by Dunbar et al. [63]. Twelve NICU’s collaborated to improve neonatal pain and sedation practices. In essence, these units developed and subsequently implemented evidence-based better practices for pain management in neonates, using such an EPIQ approach [63, 64]. At the start, all units introduced changes through plan-do-study-act cycles and verified their performance. Strategies for implementing potentially better practices varied and units identified their barriers to implementation, developed tools for improvement, and subsequently shared their experience. This approach of collaborative quality improvement techniques enhanced local quality improvement efforts and resulted in effective implementation of potentially better practices in all centers [65, 66]. Research to further improve the knowledge on pain management is obviously also needed. Using a bullet point approach, we suggest that such a research agenda covers:

- Robust pharmacodynamic outcome variables are needed [18]. The development and validation of more sophisticated pain assessment tools is needed. At present, we measure at the level of pain expression and that is not equal to pain perception.
- PK/PD information on new compounds can be generated. However, pharmacovigilance and long-term outcome data are needed after perinatal exposure to analgesics. This should include, but cannot be limited to, neurocognitive outcome [2, 7, 8, 22].
- Clinicians, ethical committees and other stakeholders should design dose-finding studies aimed at improving adequate (i.e., effective, no over- or underexposure) administration of analgesics in neonates. The animal experimental findings on neuro-apoptosis force us to reconsider drugs and doses currently administered in NICUs across the world.

## Acknowledgments 

The clinical research of K. Allegaert is supported by the Fund for Scientific Research, Flanders (fundamental clinical investigatorship 1800214N) and the research activities are further facilitated by the agency for innovation by Science and Technology in Flanders (IWT) through the SAFEPEDRUG project (IWT/SBO 130033). J. van den Anker is supported by NIH (K24DA027992, R01HD060543, U54HD071601) and the European Commission (TINN [223614], TINN2 [260908], NEUROSIS [223060]). 

## Conflict of interest 

We do not have any conflict of interest related to the topic discussed, but we do discuss off label use of drugs. 

**Table 1.** The incidence of painful procedures and its management as reported in different cohorts of (pre)term neonates. All studies collected data in the first 14 days of postnatal life [11, 12, 13].

	Rotterdam [11]	Paris [12]	Rotterdam [13]
Time interval of data collection	2001	2005 – 2006	2009
Number of patients	151	430	175
Gestational age (weeks)	32.4 (SD 4.5)	33 (SD 4.6)	31.6 (range 24 – 41)
Patient days	1,375	3,598	1,730
Number of procedures, total	19,674	42,413	21,076
Number of procedures, per day	14.3 (SD 4.0)	16 (SD 9)	11.4 (SD 5.7)
Pharmacological analgesia (%)	60.3%	57.1%	36.5%
